# Cuttlefish show flexible and future-dependent foraging cognition

**DOI:** 10.1098/rsbl.2019.0743

**Published:** 2020-02-05

**Authors:** Pauline Billard, Alexandra K. Schnell, Nicola S. Clayton, Christelle Jozet-Alves

**Affiliations:** 1 Normandie Univ, Unicaen, CNRS, EthoS, 14000 Caen, France; 2 Univ Rennes, CNRS, EthoS (Éthologie animale et humaine) - UMR 6552, F-35000 Rennes, France; 3 Comparative Cognition Lab, Department of Psychology, University of Cambridge, Cambridge CB2 3EB, UK

**Keywords:** foraging cognition, future-dependent behaviour, flexibility, cephalopods

## Abstract

Some animals optimize their foraging activity by learning and memorizing food availability, in terms of quantity and quality, and adapt their feeding behaviour accordingly. Here, we investigated whether cuttlefish flexibly adapt their foraging behaviour according to the availability of their preferred prey. In Experiment 1, cuttlefish switched from a selective to an opportunistic foraging strategy (or *vice versa*) when the availability of their preferred prey at night was predictable versus unpredictable. In Experiment 2, cuttlefish exhibited day-to-day foraging flexibility, in response to experiencing changes in the proximate future (i.e. preferred prey available on alternate nights). In Experiment 1, the number of crabs eaten during the day decreased when shrimp (i.e. preferred food) were predictably available at night, while the consumption of crabs during the day was maintained when shrimp availability was unpredictable. Cuttlefish quickly shifted from one strategy to the other, when experimental conditions were reversed. In Experiment 2, cuttlefish only reduced their consumption of crabs during the daytime when shrimps were predictably available the following night. Their daytime foraging behaviour appeared dependent on shrimps' future availability. Overall, cuttlefish can adopt dynamic and flexible foraging behaviours including selective, opportunistic and future-dependent strategies, in response to changing foraging conditions.

## 1. Introduction

Natural habitats can vary in the distribution and abundance of food availability. Many animals can navigate these environmental variations by modifying their foraging behaviour in response to the quantity and quality of food available in their environment, as well as the presence of other predators and competitors [1]. When there is ample prey, predators show selective behaviour, selectively foraging on higher quality or preferred prey and disregarding other types of food. However, when prey abundance or variety is limited, predators might exhibit opportunistic foraging, pursuing quantity more than quality [2,3]. Some species use simple cognitive mechanisms to solve such foraging problems such as responding to an environmental cue, e.g. the amount of prey diminishing. Other animals optimize their foraging behaviour through more complex cognitive mechanisms, such as enhanced spatial memory, value-based decision-making and executive control [4]. For example, predators might need to memorize food availability, when it would be optimal to eat, and where it is located. If the availability of a resource is difficult to forecast, they may need to use previous encoded knowledge about prey availability and information in the present context to facilitate foraging decisions (e.g. when and where to hunt). This decision is made on the basis of a trade-off between the cost of catching prey (e.g. energy, risk-taking) and the rewards it will provide while taking into account the probability of failing (i.e. value-based decision-making). The capacity to optimize these foraging decisions is also influenced by the capacity to restrain inappropriate motor responses, which is defined as executive control, including both inhibitory control and self-control. A lack of executive control might result in a failed attempt to capture prey immediately when the best decision might have been to stay hidden until the prey draws nearer, and thus increasing the likelihood of a successful attack.

Cuttlefish, *Sepia officinalis*, are described as opportunistic predators and exhibit a high level of diet generalism—feeding on a range of crustaceans, gastropods, fishes and other cephalopods [5,6]. Despite having a generalized diet, cuttlefish have strong individual food preferences [7–9]. They possess a large central nervous system from hatching, facilitating the ability to learn from a young age. Previous research shows that they are able to modify their behaviour in response to several distinct environments, adopting suitable and flexible mating or hunting strategies [10]. Moreover, cuttlefish are able to flexibly change their food preferences if their preferred prey is devalued (i.e. it is coated with a quinine-based solution making it bitter; [11]), and can inhibit their predatory motor behaviour when prey are visually presented but unobtainable (‘prawn-in-the-tube’ procedure, [12–16]). Cuttlefish are also capable of remembering episodic-like information based on what happened, where, and when by adjusting their foraging behaviour in response to the delay of replenishment of different food types being available [17]. Previous research suggests that episodic-like memory is linked to more complex cognitive abilities such as flexible decision-making and future planning [18,19].

In the present study, we investigate whether cuttlefish are capable of flexible decision-making by testing whether they can adjust their foraging behaviour in response to changing prey conditions. In Experiment 1 (conditions 1 and 2), we investigate whether cuttlefish are able to change their foraging behaviour in response to environmental variations (predictable availability of their preferred food item at night versus unpredictable availability), and more specifically switch between an opportunistic to a selective foraging strategy, and *vice versa*. In Experiment 2, we aim to test whether cuttlefish exhibit day-to-day flexible foraging in response to acquired knowledge about what will happen in the proximate future (availability of their preferred prey the following night).

## 2. Material and methods

### (a) Subjects

Twenty-nine sub-adult European common cuttlefish (*Sepia officinalis*) participated in this study, ranging from three to six months of age. All eggs were collected from the English Channel along the northern coast of France and the southern coast of England. Two populations of cuttlefish were used. The first population (*N* = 19) was reared at the CREC, Luc-sur-Mer, Calvados, France (49.31° N, 0.36° W). These cuttlefish were housed in individual grey plastic tanks (10 cm in diameter) with circulating natural seawater at a temperature of 15 ± 1°C and maintained under artificial light conditions (12L : 12D cycle). The second population (*N* = 10) was reared in the Marine Biological Laboratory, Woods Hole, USA (41.53° N, 70.67° W). Dorsal mantle lengths were measured (mean dorsal mantle length ± s.e.m. = 41.79 ± 1.04 mm; range = 29–58 mm). These subjects were also housed individually in plastic tanks, which were supplied with a constant flow of filtered seawater (approx. 10 l min^−1^), maintained under natural daylight conditions and at a temperature of 15–17°C. Prior to experimental trials, all cuttlefish were fed a mixed diet of food items ad libitum, including thawed frozen prawn, live grass shrimp (*Palaemonetes paludosus* and *Crangon crangon*), live gammarid shrimp (*Platorchestia platensis*) and juvenile live crabs (*Carcinus maenas* and *Hemigrapsus sanguineus*). Subjects were used in several non-invasive experiments and were housed for the remainder of their life cycle until they died following senescence.

#### (i) Food preference

For each cuttlefish, tests were conducted to determine individual food preferences between crab and shrimp. Both prey items were presented at equidistance and simultaneously to the cuttlefish. Subjects were allowed to choose one prey item only. The first prey captured by the cuttlefish was considered to be their preferred prey. Cuttlefish were tested five times per day over a period of 5 days. All subjects showed a preference for shrimp.

### (b) Experimental procedures

#### (i) Experiment 1: conditions 1 and 2

One crab was placed in each cuttlefish tank every morning. At the end of the day, we recorded whether each cuttlefish had eaten the crab, and all remaining crabs were removed from the tanks. In condition 1, one shrimp was placed in each cuttlefish tank every evening. In condition 2, one shrimp was placed in each cuttlefish tank at random. The availability or absence of the shrimp was determined by the experimenter using a random number generator (StatTrek.com). After 16 trials, we reversed the experimental conditions for cuttlefish tested in conditions 1 and 2 to assess whether cuttlefish were able to quickly and flexibility adapt their foraging strategy. In total, subjects received 16 trials in each condition (32 trials in total per individual). Trials were compacted in four blocks of four trials per condition (see electronic supplementary material, data).

#### (ii) Experiment 2

Two crabs were placed in each cuttlefish tank every morning (because these cuttlefish were older and larger and therefore required more food) at the CREC and at the MBL. At the end of the day, the number of crabs eaten was recorded for each cuttlefish, and all remaining crabs were removed from the tanks. At the end of the day, cuttlefish were provided with two shrimp every second evening (i.e. one evening out of two). Cuttlefish were tested until they reached a learning criterion of eight correct choices out of 10 consecutive trials. A choice was considered correct when cuttlefish refrained from eating the crab when shrimp were available in the evening, and when cuttlefish ate the crab when shrimp were not available in the evening.

### (c) Statistical analysis

All data were analysed with non-parametric tests and computed using R software (version 3.5.1). To test the consumption of crabs through time (i.e. blocks of four days), per condition (condition 1 versus 2), or per day for Experiment 2 (days with or without shrimp at night) we used non-parametric permutation test analyses of data from factorial experiments (aovperm function, permuco package; [20]). Effect sizes and confidence intervals were computed (see electronic supplementary material).

## 3. Results

**Figure 1.**
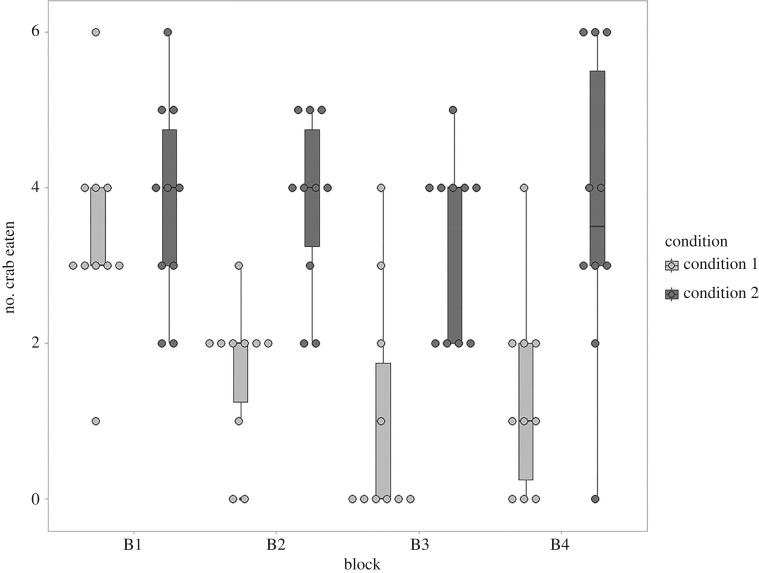
Consumption of crabs over time in conditions 1 and 2. Condition 1: consumption of crabs when shrimp were available every night. Condition 2: consumption of crabs when shrimp were only randomly available at night. The consumption of crabs significantly decreased over time in condition 1 while it was relatively stable over time in condition 2.

In Experiment 1, cuttlefish tested in condition 1 (i.e. shrimp systematically provided every night), significantly lowered their consumption of crabs during the day over time, while cuttlefish tested in condition 2 (i.e. shrimp provided at random) relatively maintained their consumption of crabs over time. The consumption of crabs was significantly different between conditions 1 and 2 (*p* < 0.001; effect size = 22.359). The effect size conveys that the variability between conditions 1 and 2 is 22 times higher than variability observed within conditions; this demonstrates a strong effect of experimental conditions on crab consumption. Cuttlefish tested in conditions 1 and 2 flexibly modified their foraging strategies when experimental conditions were reversed; demonstrated by a significant interaction between time (i.e. four blocks of 4 days) and condition (*p* = 0.030; effect size = 3.201, figure 1).

**Figure 2.**
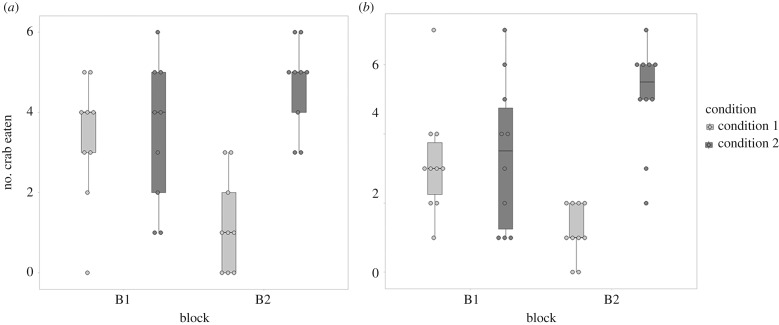
Consumption of crabs over time in Experiment 2 (i.e. shrimp were available on alternate nights). When shrimp were not available at night-time, the consumption of crabs remained stable over time in both laboratories. When shrimp were available at night-time, the consumption of crabs significantly decreased over time in both laboratories.

In Experiment 2, both cuttlefish from the CREC and from the MBL lowered their consumption of crabs during the day when shrimp were available the following night, while cuttlefish maintained their consumption of crabs during the day when no shrimp were available the following night (figure 2). Statistical analyses showed no significant effect of time (i.e. four blocks of 4 days, CREC *p* = 0.293, effect size = 1.778; MBL *p* = 0.707, effect size = 0.144) but a significant effect of the conditions (i.e. days with or without shrimp at night, CREC *p* = 0.005, effect size = 10.449; MBL *p* = 0.003, effect size = 11.737), and a significant interaction between time and conditions (CREC *p* = 0.001, effect size = 16.514; MBL *p* < 0.01, effect size = 21.962). Effect sizes for conditions and interactions were greatly above 1 (from 10 to 21 times higher), indicating that cuttlefish alter their foraging behaviour in response to the availability of shrimps the following night, and that this behavioural alteration was even more pronounced across training. Cuttlefish tested in Experiment 2 reached the learning criterion (i.e. eight correct choices out of 10 consecutive trials) in 23 ± 12 trials at the CREC and 31 ± 6 trials at the MBL.

## 4. Discussion

Our study provides evidence of flexible predatory behaviour in cuttlefish. In condition 1, when one shrimp was available every evening, cuttlefish adopted *selective* foraging behaviour, significantly reducing their consumption of crabs during the day. By contrast, in condition 2, when shrimp were available randomly through time in the evening, cuttlefish adopted an *opportunistic* foraging strategy and maintained their consumption of crabs during the day. The random availability of shrimp in this condition meant that subjects were unable to predict the availability of their preferred prey and might adopt a ‘less risky’ option of consuming crabs. This increase in crab consumption might also be the consequence of lower food supply at night. When conditions 1 and 2 were reversed cuttlefish flexibly modified their foraging behaviour. Specifically, cuttlefish that were accustomed to eating crabs during the day significantly reduced their consumption, while those who were accustomed to waiting until the evening to eat shrimp begun eating crabs during the day. Theories on risk-managing and uncertainty postulate that animals must constantly adapt to changes [21]. It has been argued that animals gather information about their proximate and distant background to reduce the uncertain outcomes of events, which is an adaptive mechanism for an organism [22,23].

In Experiment 2, both groups from the CREC and the MBL adopted a flexible foraging strategy, adjusting the consumption of their less preferred prey in response to the upcoming availability of the preferred prey the following evening. Specifically, cuttlefish ate crabs when no shrimp were available in the evening but reduced their consumption of crabs when shrimp were available in the evening. This adjustment in crab consumption cannot be explained by their nutritional state as cuttlefish were consequently eating more crabs when they had access to shrimps the previous night, and *vice versa*. Our results could be explained in terms of positive and negative anticipatory contrasts [24]. Indeed, when cuttlefish know that they will not receive any shrimp at night, they would show a positive anticipatory contrast by eating the crabs during the day in anticipation of the absence of a later reward, but when cuttlefish know that shrimp will be distributed at night, they show a negative anticipatory contrast by refraining from eating the crabs, in anticipation of receiving a later reward. This pattern suggests that cuttlefish have rapid and flexible transient foraging strategies in response to changing environmental conditions, previous experience and potentially causal knowledge. Decision-making based on expected outcomes might have been modulated by knowledge of the causal structure of the environment (i.e. if my preferred food was not provided the previous night, I will have access to shrimp the following night).

But are the dynamic foraging patterns in cuttlefish driven by future-oriented behaviours or planning? According to the definition of future planning in animals [18], the observed behaviour must be flexible and sensitive to its consequences (e.g. [25]). Our study shows that cuttlefish are capable of adjusting their foraging behaviour day-to-day in response to proximate-future environmental conditions (i.e. future-dependent foraging). Moreover, the decision they make during the day (i.e. the decision to eat the crabs or not) will likely have an impact on their later motivation to eat the shrimp in the evening. If cuttlefish decide to eat the crabs, then their motivation to eat the shrimp in the evening might be lowered, and they might ‘miss’ an opportunity to eat their preferred prey. However, at this stage, we cannot validate whether this future-dependent foraging behaviour observed in cuttlefish is underpinned by their ability to plan for the future. In order to determine whether cuttlefish foraging behaviour qualifies as future planning, we still need to test one critical criterion—are cuttlefish behaving independently of their current motivational state (i.e. desire to eat shrimps in the present moment)? Nevertheless, these results represent a promising way for further studies on flexibility and future-oriented behaviour in cephalopods. Given that cephalopods diverged from the vertebrate lineage approximately 550 million years ago, finding comparable future-oriented abilities in cuttlefish might provide valuable evolutionary insight into the origins of such a complex cognitive ability.

## Supplementary Material

Raw data and confidence intervals

## Data accessibility

All data are available from the Dryad Digital Repository: https://doi.org/10.5061/dryad.5tb2rbp0z [26].
