# Supplementary material for: Cuttlefish show flexible and future-dependent foraging cognition
Source: Biol Lett. 2020 Feb 5;16(2):20190743. doi: 10.1098/rsbl.2019.0743 (PMC7058941; doi:10.1098/rsbl.2019.0743)

Supplementary material:

- Condition 1 & 2

aovperm(NB_CRAB~CONDITION*BLOCK+Error(IND/CONDITION*BLOCK),data=stim,np=100000)

| CONDITION | BLOCK | NB_CRAB | IND | ORDER |
| --- | --- | --- | --- | --- |
| Condition 1 | B1 | 4 | 1 | 1 |
| Condition 1 | B1 | 4 | 2 | 1 |
| Condition 1 | B1 | 3 | 4 | 1 |
| Condition 1 | B1 | 1 | 5 | 1 |
| Condition 1 | B1 | 3 | 8 | 1 |
| Condition 2 | B1 | 4 | 9 | 1 |
| Condition 2 | B1 | 2 | 10 | 1 |
| Condition 2 | B1 | 4 | 12 | 1 |
| Condition 2 | B1 | 4 | 14 | 1 |
| Condition 2 | B1 | 3 | 16 | 1 |
| Condition 1 | B2 | 2 | 1 | 1 |
| Condition 1 | B2 | 2 | 2 | 1 |
| Condition 1 | B2 | 3 | 4 | 1 |
| Condition 1 | B2 | 2 | 5 | 1 |
| Condition 1 | B2 | 1 | 8 | 1 |
| Condition 2 | B2 | 4 | 9 | 1 |
| Condition 2 | B2 | 3 | 10 | 1 |
| Condition 2 | B2 | 4 | 12 | 1 |
| Condition 2 | B2 | 4 | 14 | 1 |
| Condition 2 | B2 | 4 | 16 | 1 |
| Condition 1 | B3 | 3 | 1 | 1 |
| Condition 1 | B3 | 1 | 2 | 1 |
| Condition 1 | B3 | 0 | 4 | 1 |
| Condition 1 | B3 | 0 | 5 | 1 |
| Condition 1 | B3 | 0 | 8 | 1 |
| Condition 2 | B3 | 2 | 9 | 1 |
| Condition 2 | B3 | 2 | 10 | 1 |
| Condition 2 | B3 | 4 | 12 | 1 |
| Condition 2 | B3 | 4 | 14 | 1 |
| Condition 2 | B3 | 2 | 16 | 1 |
| Condition 1 | B4 | 1 | 1 | 1 |
| Condition 1 | B4 | 0 | 2 | 1 |
| Condition 1 | B4 | 2 | 4 | 1 |
| Condition 1 | B4 | 2 | 5 | 1 |
| Condition 1 | B4 | 0 | 8 | 1 |
| Condition 2 | B4 | 0 | 9 | 1 |
| Condition 2 | B4 | 2 | 10 | 1 |
| Condition 2 | B4 | 3 | 12 | 1 |
| Condition 2 | B4 | 3 | 14 | 1 |
| Condition 2 | B4 | 3 | 16 | 1 |
| Condition 2 | B1 | 5 | 1 | 2 |
| Condition 2 | B1 | 6 | 2 | 2 |
| Condition 2 | B1 | 5 | 4 | 2 |
| Condition 2 | B1 | 3 | 5 | 2 |
| Condition 2 | B1 | 2 | 8 | 2 |
| Condition 1 | B1 | 3 | 9 | 2 |
| Condition 1 | B1 | 3 | 10 | 2 |
| Condition 1 | B1 | 6 | 12 | 2 |
| Condition 1 | B1 | 4 | 14 | 2 |
| Condition 1 | B1 | 3 | 16 | 2 |
| Condition 2 | B2 | 2 | 1 | 2 |
| Condition 2 | B2 | 5 | 2 | 2 |
| Condition 2 | B2 | 5 | 4 | 2 |
| Condition 2 | B2 | 2 | 5 | 2 |
| Condition 2 | B2 | 5 | 8 | 2 |
| Condition 1 | B2 | 0 | 9 | 2 |
| Condition 1 | B2 | 2 | 10 | 2 |
| Condition 1 | B2 | 2 | 12 | 2 |
| Condition 1 | B2 | 2 | 14 | 2 |
| Condition 1 | B2 | 0 | 16 | 2 |
| Condition 2 | B3 | 5 | 1 | 2 |
| Condition 2 | B3 | 2 | 2 | 2 |
| Condition 2 | B3 | 4 | 4 | 2 |
| Condition 2 | B3 | 4 | 5 | 2 |
| Condition 2 | B3 | 4 | 8 | 2 |
| Condition 1 | B3 | 0 | 9 | 2 |
| Condition 1 | B3 | 0 | 10 | 2 |
| Condition 1 | B3 | 4 | 12 | 2 |
| Condition 1 | B3 | 2 | 14 | 2 |
| Condition 1 | B3 | 0 | 16 | 2 |
| Condition 2 | B4 | 6 | 1 | 2 |
| Condition 2 | B4 | 4 | 2 | 2 |
| Condition 2 | B4 | 6 | 4 | 2 |
| Condition 2 | B4 | 6 | 5 | 2 |
| Condition 2 | B4 | 4 | 8 | 2 |
| Condition 1 | B4 | 0 | 9 | 2 |
| Condition 1 | B4 | 2 | 10 | 2 |
| Condition 1 | B4 | 1 | 12 | 2 |
| Condition 1 | B4 | 4 | 14 | 2 |
| Condition 1 | B4 | 1 | 16 | 2 |

- Experiment 2
  - CREC

aovperm(NB_CRAB~CONDITION*BLOCK+Error(IND/CONDITION*BLOCK),data=moi,np=100000)

| IND | NB_CRAB | CONDITION | BLOCK |
| --- | --- | --- | --- |
| A6 | 0 | C1 | B1 |
| A9 | 4 | C1 | B1 |
| A10 | 3 | C1 | B1 |
| B2 | 5 | C1 | B1 |
| G41 | 4 | C1 | B1 |
| G45 | 2 | C1 | B1 |
| G46 | 5 | C1 | B1 |
| G47 | 4 | C1 | B1 |
| G48 | 3 | C1 | B1 |
| A6 | 1 | C2 | B1 |
| A9 | 1 | C2 | B1 |
| A10 | 2 | C2 | B1 |
| B2 | 5 | C2 | B1 |
| G41 | 4 | C2 | B1 |
| G45 | 3 | C2 | B1 |
| G46 | 6 | C2 | B1 |
| G47 | 4 | C2 | B1 |
| G48 | 5 | C2 | B1 |
| A6 | 1 | C1 | B2 |
| A9 | 0 | C1 | B2 |
| A10 | 0 | C1 | B2 |
| B2 | 3 | C1 | B2 |
| G41 | 2 | C1 | B2 |
| G45 | 0 | C1 | B2 |
| G46 | 1 | C1 | B2 |
| G47 | 3 | C1 | B2 |
| G48 | 1 | C1 | B2 |
| A6 | 5 | C2 | B2 |
| A9 | 5 | C2 | B2 |
| A10 | 3 | C2 | B2 |
| B2 | 6 | C2 | B2 |
| G41 | 5 | C2 | B2 |
| G45 | 3 | C2 | B2 |
| G46 | 4 | C2 | B2 |
| G47 | 6 | C2 | B2 |
| G48 | 5 | C2 | B2 |

- - MBL

aovperm(NB_CRAB~CONDITION*BLOCK+Error(IND/CONDITION*BLOCK),data=alex,np=100000)

| IND | NB_CRAB | CONDITION | BLOCK |
| --- | --- | --- | --- |
| F1 | 3 | C1 | B1 |
| F2 | 3 | C1 | B1 |
| F3 | 1 | C1 | B1 |
| F4 | 7 | C1 | B1 |
| F5 | 2 | C1 | B1 |
| F6 | 3 | C1 | B1 |
| F7 | 4 | C1 | B1 |
| F8 | 2 | C1 | B1 |
| F9 | 4 | C1 | B1 |
| F10 | 3 | C1 | B1 |
| F1 | 1 | C2 | B1 |
| F2 | 4 | C2 | B1 |
| F3 | 1 | C2 | B1 |
| F4 | 7 | C2 | B1 |
| F5 | 1 | C2 | B1 |
| F6 | 5 | C2 | B1 |
| F7 | 6 | C2 | B1 |
| F8 | 2 | C2 | B1 |
| F9 | 4 | C2 | B1 |
| F10 | 3 | C2 | B1 |
| F1 | 1 | C1 | B2 |
| F2 | 1 | C1 | B2 |
| F3 | 0 | C1 | B2 |
| F4 | 2 | C1 | B2 |
| F5 | 1 | C1 | B2 |
| F6 | 2 | C1 | B2 |
| F7 | 0 | C1 | B2 |
| F8 | 1 | C1 | B2 |
| F9 | 2 | C1 | B2 |
| F10 | 2 | C1 | B2 |
| F1 | 5 | C2 | B2 |
| F2 | 5 | C2 | B2 |
| F3 | 3 | C2 | B2 |
| F4 | 7 | C2 | B2 |
| F5 | 5 | C2 | B2 |
| F6 | 6 | C2 | B2 |
| F7 | 6 | C2 | B2 |
| F8 | 6 | C2 | B2 |
| F9 | 2 | C2 | B2 |
| F10 | 6 | C2 | B2 |

Confidence intervals

- Conditions 1 & 2

plot(aovperm(NB_CRAB~CONDITION*BLOCK+Error(IND/CONDITION*BLOCK),data=stim,np=100000))


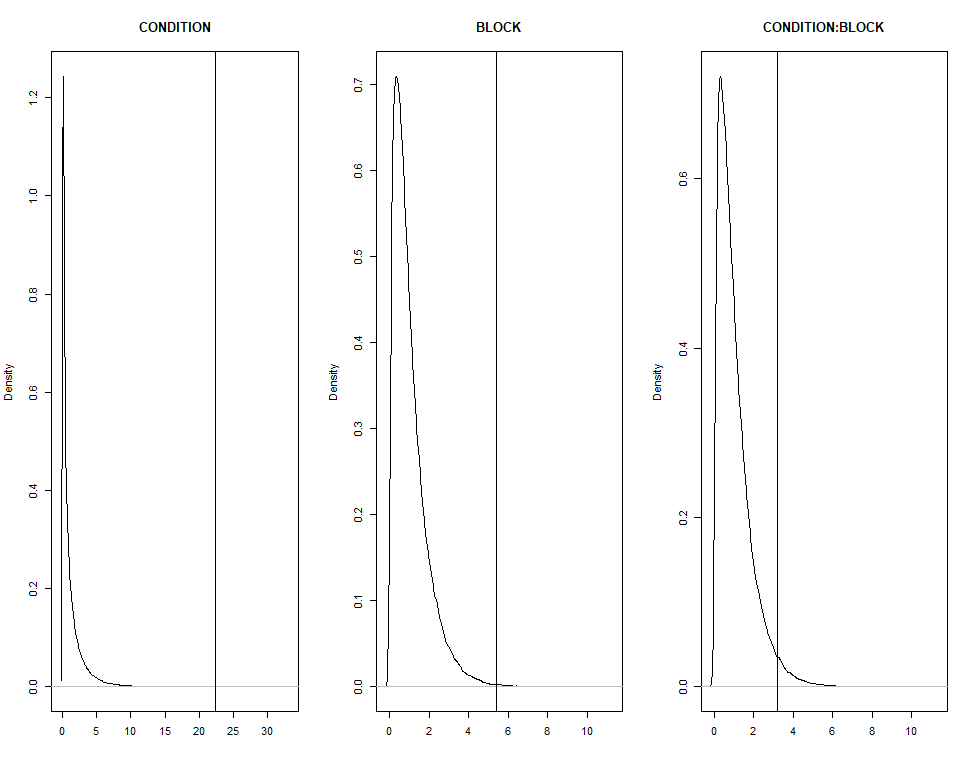


- Experiment 2
  - CREC

Plot(aovperm(NB_CRAB~CONDITION*BLOCK+Error(IND/CONDITION*BLOCK),data=moi,np=100000))


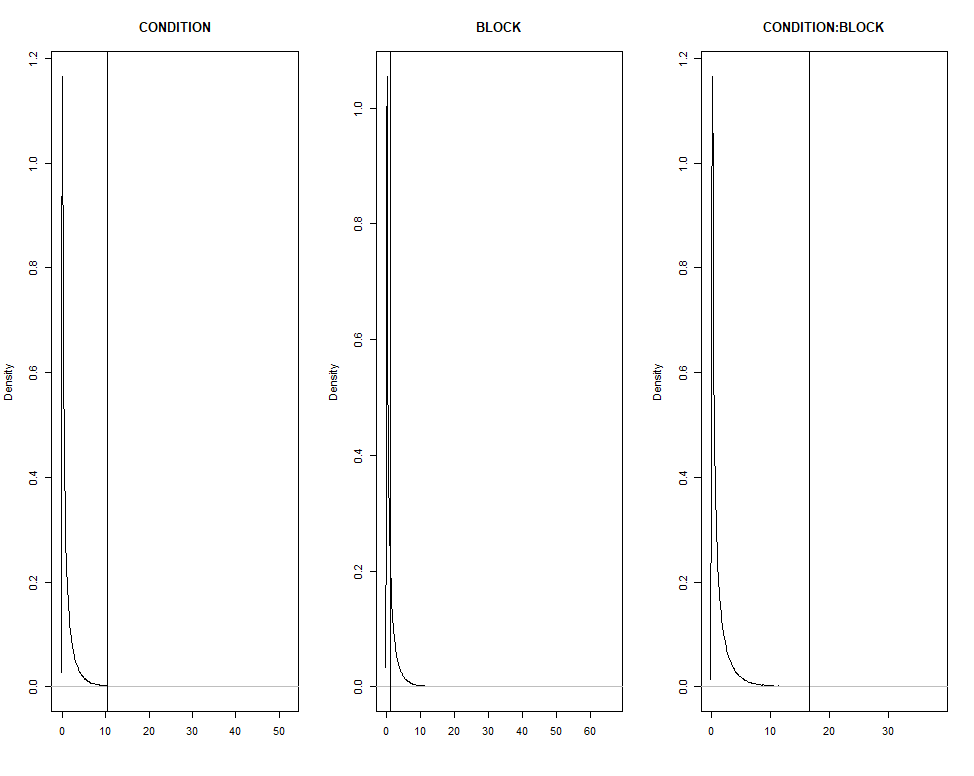


- - MBL

plot (aovperm(NB_CRAB~CONDITION*BLOCK+Error(IND/CONDITION*BLOCK),data=alex,np=100000))


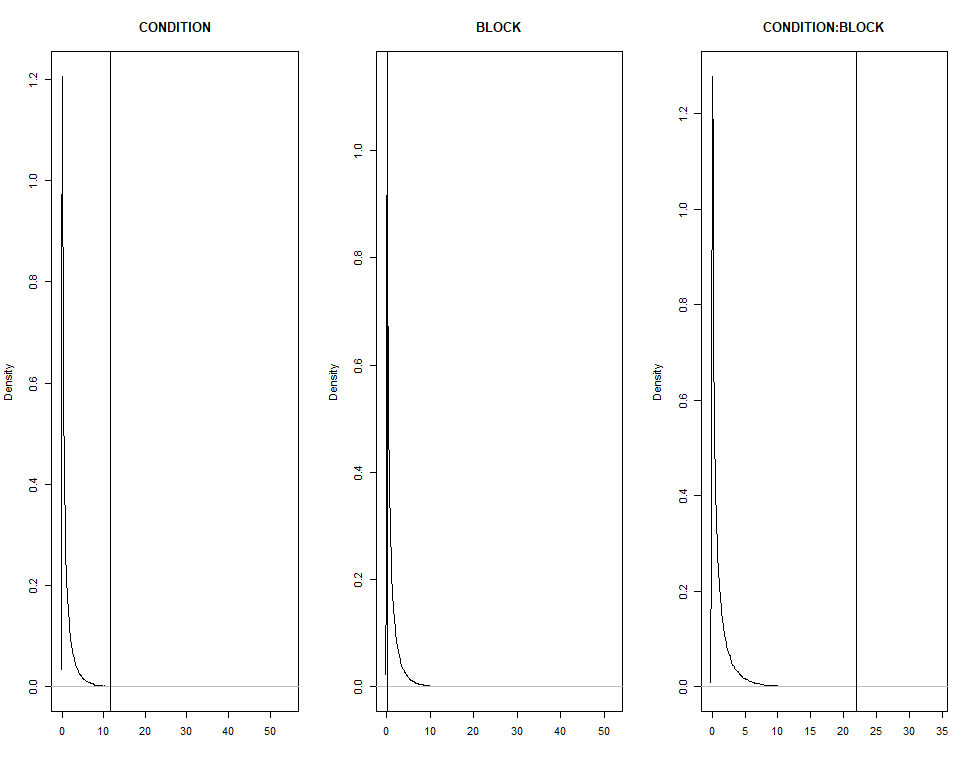

Supplement: Raw data and confidence intervals [file rsbl20190743supp1.docx]
